# All That Glitters Is Not Gold: The Indian Healthcare System

**DOI:** 10.7759/cureus.39892

**Published:** 2023-06-02

**Authors:** Venkataramana Kandi

**Affiliations:** 1 Clinical Microbiology, Prathima Institute of Medical Sciences, Karimnagar, IND

**Keywords:** pandemic, coronavirus disease-19 (covid-19), global healthcare systems, illiteracy, indian healthcare system, public-private partnerships, healthcare systems, healthcare

## Abstract

Healthcare is the most essential requirement for a better quality of life. Governments throughout the world ensure the establishment of improved healthcare systems that are on par with global healthcare systems for people, irrespective of their socioeconomic situation. It is crucial to understand the status of healthcare establishments existing in a country. The coronavirus disease 2019 (COVID-19) pandemic posed an imminent challenge concerning the quality of healthcare in various countries throughout the world. There were different types of problems faced by most nations irrespective of their socioeconomic status and financial capabilities. India also struggled to cope with the initial times of the COVID-19 pandemic wherein the hospitals were overwhelmed with patients and limited infrastructural capabilities, resulting in considerable morbidity and mortality. The greatest achievement of the Indian healthcare system was to increase access to healthcare by encouraging private players and boosting public-private partnerships to deliver better healthcare to people. Moreover, the Indian government ensured healthcare access to people from rural areas by establishing teaching hospitals. However, the major drawback in the Indian healthcare system appears to be illiteracy among people and exploitation by healthcare stakeholders that include physicians, surgeons, pharmacists, and capitalists, including hospital management and pharmaceutical industries. Nevertheless, like two sides to a coin, the Indian healthcare system has both pros and cons. The limitations in the healthcare system need to be addressed to improve the quality of healthcare provided to people in general and especially during disease outbreaks similar to the COVID-19 pandemic.

## Editorial

India is a developing nation with a hugely growing population that has recently overtaken the Chinese population by numbers as reported recently by the United Nations [1]. Approximately 70% of Indians live in rural areas, pursuing farming and agriculture for a living, which is the main source of income. It was reported that a majority of the people living in the rural parts of India earn around one dollar, which puts them in the poverty category as described by international standards. The poor living conditions in rural India predispose them to communicable and non-communicable diseases. The major cause for the spread of communicable diseases is unfavorable housing, illiteracy, poor sanitation, and hygiene among others. The non-communicable diseases like asthma (13% of the global burden) and malnutrition arise from the burning of wood and other farm residuals and lack of knowledge of nutritional requirements, respectively. Urban living also is plagued by high population density, lack of potable water supply, and improper waste management among others that could, in turn, predispose them to communicable diseases. Additionally, people living in urban areas suffer from severe stress related to work, and dormant lifestyles that may predispose them to non-communicable diseases, including cardiovascular diseases and kidney diseases among others [2].

Moreover, health and education facilities in the rural parts of India appear to be defective/flawed. This is due to the fact that doctors and teachers who are posted in rural areas do not accept the job or look for a transfer to an urban setup. Additionally, hospitals in rural parts are not properly equipped, thereby discouraging specialist doctors from working. People living in rural areas are grossly uneducated and poor and do not believe in allopathic medicine. They prefer treatment by local, uneducated, and uncertified persons.

The Government of India (GOI) proposed a National Health Mission (NHM) that included two branches named National Rural Health Mission (NRHM), and National Urban Health Mission (NUHM) in the years 2004-2005 and 2012, respectively. This was introduced to integrate primary (village hospitals), secondary (district hospitals), and tertiary (medical colleges and attached teaching hospitals) healthcare facilities in rural and urban settings [3].

A revised national health policy (NHP) was instituted in 2017 under Prime Minister Narendra Modi's government, which had majorly tried to address the issues of increasing healthcare costs and the rising burden of non-communicable and communicable diseases [4]. NHP aimed to provide universal and good quality healthcare for people of all ages and different financial backgrounds. Additionally, the NHP was devised according to the Sustainable Development Goals (SDGs) that recommend global strategies for improved healthcare access at affordable and low costs. However, the NHP was optional, and the state governments were not compelled to implement this scheme [3]. Under the revised NHP of 2017, the major goals for health included the elimination of leprosy (2018), kala-azar (2017), and tuberculosis. Interestingly, none of these goals had been realized nor there is any realistic possibility of the elimination of tuberculosis by 2025. Moreover, the health indicators have revealed that the disease burden has doubled in India despite a decrease in mortality rates. Also, Indian health policies are greatly influenced by health inequities like income inequities, occupational inequities, educational inequities, rural, urban, and interstate inequities, and religious, caste, and tribal inequities [3].

The major achievements of NHP of India are the introduction and implementation of the Swachh Bharat Mission (2014-19) and Ayushman Bharat Yojana/Pradhan Mantri Jan Arogya Yojana (National Health Protection Mission). The Swachh Bharat Mission was instrumental in promoting health and environmental hygiene among people. The Ayushman Bharat Yojana, which was introduced in 2018, was aimed to provide medical insurance policies to poor people. Additionally, the Indian NHP also took initiatives like Sarwa Shiksha Abhiyan (education for all), National AYUSH Mission (alternative/complementary medicine), and Affordable Medicines and Reliable Implants for Treatment (AMRIT) among other schemes [5].

India works closely with the World Health Organization (WHO), United Nations Children’s Fund (UNICEF), World Trade Organization (WTO), Bill and Melinda Gates Foundation (BMGF), Global Alliance of Vaccines and Immunization (GAVI), and the Global Fund to fight AIDS, Tuberculosis, and Malaria, and the World Bank and Asian Development Bank for the implementation of health policies [3]. India has seen an increase in life expectancy at birth (LEB); 69.6 years in 2020 from 47.74 years in 1970. However, the life expectancy of Indians is lower compared to other middle-income countries like Brazil (74 years), China (75 years), and Srilanka (74 years) among others. This could be due to the availability of medicines and better healthcare facilities. Besides, the LEB had gross interstate variability. This suggests non-uniformity of the healthcare services within the same country that could affect the long-term national health plans. Anganwadi centers were established under the Integrated Child Development Services (ICDS) program at the village level to ensure that women are educated and that pregnant women and newborns were provided with nutritional supplements, which could have been instrumental in lowering infant mortality rates (IMR) and low birth weight babies [3].

Additionally, it was identified that educating the females about the risks associated with low-age pregnancies, thereby increasing the maternal childbearing age and enhancing the birth spacing (between two successive pregnancies) was instrumental in lowering the IMR in India [6].

Access to healthcare among people was increased with the establishment of teaching hospitals, especially in rural areas. This was aimed to provide tertiary care to the public and limit people traveling to distant places for such care. However, this has not completely benefited local people since many doctors working in such institutions either abstain from duty and work for limited hours or refer the same patients to visit their private clinics for financial gains.

Despite the current state government trying its best to improve the infrastructure in rural hospitals, it has not encouraged the doctors to work there due to low salaries and fewer earning opportunities through supplementary private practice. Nothing much has changed despite a steep increase in remunerations probably owing to poor government monitoring.

The local governments are encouraged to implement their own health initiatives alternative to those suggested by the central GOI. The Telangana State Government (TSG) instituted a medical insurance scheme known as "Arogyasri", an alternative to Ayushman Bharat Yojana/Pradhan Mantri Jan Arogya Yojana that benefits poor and low-income families [7]. However, due to the prevailing perception that government hospitals are inferior to private establishments, many people prefer to get healthcare from private hospitals. This has led to the exploitation of the people by these organizations.

The TSG encouraged private healthcare establishments by allowing them to provide patient care under the "Arogyasri" program. However, private hospitals have been misusing this by creating false medical records and fake patients to claim compensation from the government for the treatment they claim to have provided to the patients through the "Arogyasri" program.

The major reason for such misappropriation is the lack of proper government monitoring and corruption among government officials. Additionally, the for-profit commercially inclined private sector happens to manage higher than 50% of the healthcare industry [3]. Besides, it appears that the medical insurance companies and the hospitals have been misusing the insurance policies of the people by preparing fake patient records as proof of medical care that includes the treatment and management of various diseases including elective surgeries. This contributes to the unnecessary financial burden on the governments and increased exploitation by private healthcare establishments like hospitals.

The GOI-enforced Clinical Establishment Act (CEA) in the year 2010 was aimed at regulating all clinical establishments, including hospitals and laboratories. The CEA was implemented to enable uniformity of healthcare quality provided and ensure minimum standards in the diagnosis and treatment of patients [8].

The coronavirus disease 2019 (COVID-19) pandemic has been controlled in India through a strict lockdown during the initial days. This was inevitable because the delicate healthcare infrastructure in India could not have borne the high burden of infected patients. Moreover, illiterate people could have been increasingly affected due to ignorance, which might have changed the course of the pandemic to the worst outcomes in India. Other factors that affected the healthcare system during the COVID-19 pandemic included a shortage of skilled human resources/manpower, a low doctor-to-patient ratio, and a rising burden of NCDs and infectious diseases among others [9].

Conversely, the Accredited Social Health Activists (ASHAs), who are trained community healthcare assistants under the NRHM, were instrumental in the improvement of healthcare delivery, especially at the community level [4]. The ASHAs workforce was utilized for the effective implementation of the national immunization programs and COVID-19 vaccine delivery during the pandemic.

The diagnostic capabilities of the novel severe acute respiratory syndrome coronavirus-2 (SARs-CoV-2) infection were severely limited during the initial period of the pandemic, wherein most tertiary care centers throughout the country were not adequately equipped. The GOI had made it mandatory for all tertiary care centers and rural teaching hospitals to establish accredited molecular laboratories for the diagnosis of SARS-CoV-2 infection. However, genomic sequencing capabilities are very limited even today, which may influence the global epidemiological surveys that study the prevailing viral variants. Moreover, the accreditation of medical establishments in India is not mandatory, and this could be the reason for the lack of quality, efficiency, and reliability. India has independent assessment and accreditation agencies for hospitals and laboratories, including National Accreditation Board for Hospitals & Healthcare Providers (NABH) and National Accreditation Board for Testing & Calibration Laboratories (NABL), respectively [10].

**Figure 1:**
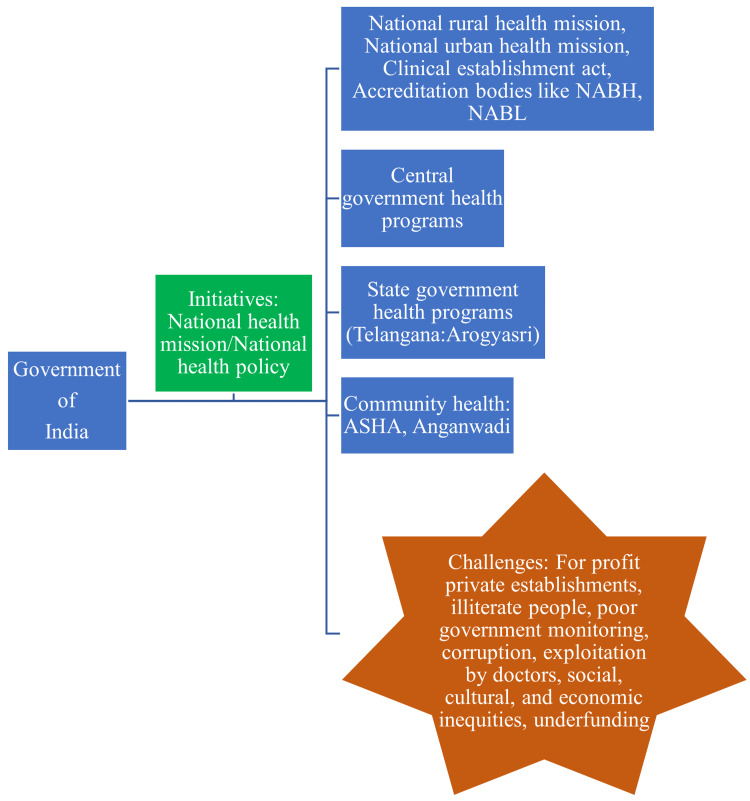
The health initiatives and challenges in India Image credit: Venkataramana Kandi NABH: National Accreditation Board for Hospitals & Healthcare Providers; NABL: National Accreditation Board for Testing & Calibration Laboratories; ASHA: Accredited Social Health Activist

Another significant challenge of the Indian healthcare system is the lack of a robust reporting system for seasonal, communicable, and non-communicable diseases, which severely affects the government's planning system [11]. The accountability, consistency, accuracy, and reliability of electronic Health Management Information Systems (HMIS) appear to be questionable and require strengthening strategies. Additionally, the implementation of national and state-level health programs is severely affected by underfunding due to financial constraints. The Indian government's health initiatives and potential challenges are depicted in Figure 1.

In light of these observations, the governments must be more vigilant towards operational activities of healthcare systems run by them as well as increase the auditing of for-profit private healthcare establishments and strengthen the government-run healthcare institutions. It is the responsibility of respective government administrations to educate people about prevalent communicable and non-communicable diseases and preventive measures. The COVID-19 pandemic must be considered a lesson and governments must ensure better preparedness to tackle such health emergencies in the future. Governments need to take initiatives to minimize and eliminate social, cultural, and economic inequities and promote universal health. Additionally, increased funding, improved infrastructure, better education, and training would be instrumental in providing superior public healthcare services.
